# A practical framework to foster climate action through sport for development and peace

**DOI:** 10.3389/fspor.2025.1642492

**Published:** 2025-08-21

**Authors:** Alessio Norrito, Yvanna Todorova

**Affiliations:** ^1^ Faculty of Business & Law, Manchester Metropolitan University, Manchester, United Kingdom; ^2^ School of Sport, Exercise, and Health Sciences, Loughborough University, Loughborough, United Kingdom

**Keywords:** sport for development and peace, climate action, game design, ecological emotions, mechanics dynamics aesthetics

## Abstract

The climate crisis necessitates innovative approaches to foster ecological emotions and motivate pro-environmental action, particularly among young people. This conceptual paper explores how the Sport for Development and Peace (SDP) sector can more effectively address this challenge by drawing insights from game design theories. We propose the Mechanics-Dynamics-Aesthetics (MDA) framework, as a heuristic tool for intentionally designing SDP interventions. By starting with desired ecological emotions (Aesthetics), practitioners can shape play-based interactions (Dynamics) through carefully chosen rules and resources (Mechanics). This approach aims to cultivate deeper environmental empathy and encourage climate action by structuring embodied experiences within SDP. We theorize and explain how SDP can be designed to be an imaginative space of learning. In doing so, the paper addresses a significant necessity of implementing SDP initiatives that foster climate action. Finally, we encourage researchers to cross disciplinary boundaries and adopt theoretical imagination in addressing rapidly evolving social challenges.

## Introduction

The most urgent and all-encompassing contemporary challenge is the climate crisis (1), defined by the global increase in temperatures and the deterioration of the natural environment of planet Earth (2). The climate crisis is not only affecting planetary systems, but has also profound effects on human well-being (1, 3), significantly impacting both practical access to sustenance resources across the globe (4), as well as mental health (5).

Specifically focusing on mental health, the climate crisis has been the source of a series of emotions that can negatively affect well-being. These emotions, which characterize the interplay between humans and the environment and which we term *ecological emotions*, can have both relatively negative and positive connotations. Negative emotions manifest in specific forms in relation to the environment, such as eco-anxiety, yet ultimately relate to wider aspects of grief, distress, anxiety, guilt, and anger (6, 7). Positive emotions, on the other hand, may refer to hope, connection, awe, and topophilia [a sense of bonding between people and places; (8)]. While the sentiment towards the climate crisis is generally negative, positive emotions still exist and can provide an avenue for change. As the divide between positive and negative emotions can be quite complex and reductive (8), this paper argues that ecological emotions ultimately shape our relationship with the environment, and, when these emotions “stick” to form a collective sense of urgency (9, 10), they can motivate both collective and individual climate action.

Sport and leisure have both been deemed as highly affective spaces (11). Both are areas where the climate crisis can manifest and, as a consequence, climate action can potentially thrive (12, 13). For example, within the field of Sport for Development and Peace (SDP), which aims to use sport as a vehicle to achieve wider objectives of development, the role of sport in addressing the climate crisis and fostering climate action is increasingly being discussed (14–16). Within both spaces, we see the concept of “play” holding a key central role in both fostering positive emotions and providing avenues for change. However, the role of play concerning emotions and climate action requires further investigation, particularly within the SDP field (17, 18).

Indeed, competition, dominance, and othering are also significant characterizing aspects of sport, that can be dissonant with concepts of fun, play, and collective solidarity more broadly (19). Moreover, sport both contributes to and is affected by climate degradation (20, 21). In this conceptual contribution, we seek to explore how SDP practice can foster ecological emotions to positively influence climate action and eco-friendly behaviours. We do so because the SDP sector, while increasingly aware of a need to address climate change through its practices (14, 16), lacks a practical strategy to do so. Specifically, we conceptually advance an intentional framework for designing programs that specifically target ecological emotions, foster collective empowerment, and lead to collective and individual climate action.

To help the SDP sector intentionally design for ecological empathy and foster climate action, we explore what mechanisms could be implemented to build empathy with the natural environment, and in turn help participants learn and act in support of the planet. To do so, we propose the Mechanics-Dynamics-Aesthetics (MDA) framework, originating from game design (22, 23), as a valuable heuristic tool for designing and analyzing SDP interventions. The paper argues that this framework offers a novel way to intentionally structure embodied experiences within sport to achieve affective outcomes, while advocating for transdisciplinary cooperation to improve current SDP practices (24).

### Emotions and nature: theoretically grounding an individual and relational crisis

Starting from this necessity of transdisciplinary engagement, to pinpoint the importance of the environment towards embodied well-being, we must start with the inherent physiological effects that nature has on our emotions. Indeed, the relationship between humans and their environment is exemplified by our relationship to nature. Human psychophysiological systems have evolved in the context of the natural world. Therefore, nature provides an optimum setting for healthy emotional regulation. The mass migration to urban cities disrupted this by introducing a new age of increasing distance from nature (25). This is particularly reflected in the new generation of young people who are reporting low levels of connectedness to nature (26), and is especially important in the context of ecological emotions. With the increasing distance from nature, there is a lower connection of what is at stake when it comes to the climate crisis and the need for climate action (27, 28).

Outside of this, nature also has implications for human health and wellbeing. The removal of humans from their ancestral environment to a more artificial environment (i.e., urban areas) has also been accompanied by additional challenges to health such as sedentary behaviour (29, 30), environmental pollutants (31), and emotional dysregulations (32). The idea that spending time in nature can improve health and wellbeing has been recorded as early as the 1800s (33), however, in more recent years, experimental work has sought to understand this relationship better. Consistently, empirical studies demonstrate that spending time in nature, as opposed to urban settings, is important for emotional regulation (34). Furthermore, spending time in nature can increase pro-environmental behaviours (35, 36), lower rumination [defined as fixating on negative thoughts; (37)] and reduce loneliness (38). Therefore, the climate crisis poses a threat to the psychophysiological wellbeing of humans as well as to planetary systems. As the influence of technology grows, virtual nature interventions have become an increasingly appealing avenue for connecting people with nature to improve mental health and pro-environmental behaviours (39).

Further exploring disciplines and moving across silos of knowledge, cultural sociology tells us that the increasing physical distance between people and nature has led to an increase in the affective bond of humans towards the environment. Indeed, when humans grieve the loss of natural or built environment, we refer to this emotion as solastalgia (40). As we have earlier defined, we also have topophilia, an emotion referring to the love of people towards places, yet a sometimes ambiguous and contradictory love. In their foundational work, Tuan (8) explains how people see in nature a refuge, a teacher, and a source of beauty, both fearing and idealizing the natural environment. The ideas of solastalgia and topophilia interestingly complement the psychophysiological needs of humans, with their recent introduction in the English language telling us that such higher connection to nature is qualitatively complex, both at an individual and collective level. Indeed, we can see that emotions are not just private and internal feelings, but, just like for the “creation” of topophilia and solastalgia, they are socially and politically shaped by our interactions with others and with the world (10).

Ahmed (10) further argues in her monograph that emotions influence collective identities, and that the objects of emotions are shaped as an effect of their circulation in society. To put simply and in the context of our study, the climate crisis exists because people have collectively realized a danger in the deterioration of the environment through their emotional engagement. Their feelings of fear, grief, regret, but also hope, towards the environment have in turn socially and politically shaped the climate crisis that we now seek to globally address. And these emotions have risen due to the constant and repeated exposure of humans to the symptoms of climate change. Ahmed (9) further defines this constant exposure as “stickiness”, the idea that objects accumulate emotional meanings through repetitions. The more we see a negative change in the environment, the more the “crisis” element of climate change amplifies and emotionally resonates (6, 8). Similarly, the more people care about the environment, the more others will be inclined to care (41). For this reason, within this conceptual study, we see emotions as relational, active forces that can shape both meaning and action.

### Sport for development and peace—issues by design?

Within this context, sport presents a quintessential example of an embodied practice where individuals connect with their physical surrounding. Aspects such as the air quality, the weather, or the provision of appropriate spaces, all affect both the possibility and the enjoyment of a given sport. These aspects have led to a creation of a new subsdiscipline within the domain sport management and sociology, sport ecology, which refers to the bidirectional relationship between sport and the environment (13, 42). The degradation of a specific environmental attribute may mean the loss of a sport. A clear example lies in the questionable long-term sustainability of the Winter Olympics amidst global warming, and of winter sports more generally (43). This impending feeling of loss towards a sport not only makes environmental change more salient, but also emotionally resonant (44, 45). Nonetheless, studies show that the role of sport is not exclusively that of signifying climate change, but it can also be a space in which to foster place attachment and environmental sensitivity. For example, Amann & Doidge (12) discuss how football can be a space to mobilise fans on the topic of climate change, providing an avenue for positive collective behaviour and social change. Sport can also be used as a vehicle to educate and engage disadvantaged communities on the topic of environmental preservation (46). Similarly, environmental activism can generate from athletes themselves (47), which can be a powerful voice to inspire climate action. Among these efforts, SDP is emerging as a sport-based space in which to address the climate crisis (14, 15, 48).

As we have earlier mentioned, SDP is a movement that purports the use of sport as a vehicle to achieve wider development goals (49, 50). While the movement is becoming increasingly professionalized and institutionalized (51), its foundation lies in the value of sport as a global game and form of play that can inspire change and development. This belief however does not come without critiques of evangelism, whereby a mythopoeic value is attributed to sport as a simplistic cure for complex social problems (52, 53) or where, in some cases, sport may be enmeshed within existing social problems (54). To combat such belief, researchers suggest that sport works towards social change only under specific contexts and mechanisms, which are situationally dependent and require monitoring and evaluation (55). Under the right circumstances sport can indeed promote health and social goals, positively engaging communities and providing educational value for youth. These circumstances often relate to the idea of play in SDP, rather than the sport element more strictly, given that sport can also be a source of competition and conflict, fostering othering and heightening divisions (17, 18, 56).

It is indeed within this balance of sport and play that we can advance a further critique of SDP when we address the topic of climate action and environmental emotions. As it currently stands, scholars view SDP as an ecological endeavour (14), while also focusing on greening SDP practices themselves (16). However, the frequent rigidity within many SDP programs, often prioritizing rule-setting, discipline, and the prevention of antisocial behaviour, can unduly limit exploration, curiosity, and freedom (19). Recent calls indeed highlight the need for SDP to engage with emotions (54, 57–59). Along broader critical lines, sport often promotes an anthropocentric extractive view of nature, treating the physical environment as a space to be consumed rather than cared for (60, 61). Within the case of SDP specifically, initiatives can be individualistic and reproduce neoliberal values, failing to foster the collective agency needed for meaningful change (19, 62).

Addressing these critiques, we propose a practical framework for designing programs that specifically target the nurturing of ecological emotions, and that foster collective empowerment.

### Designing for emotions: the mechanics-dynamics-aesthetics (MDA) framework

Within the SDP movement, several theories and frameworks have been used both to guide, monitor, and evaluate SDP programmes and initiatives. These range from wider theories, such as the context-mechanism-outcomes model of critical realism (63), to specific sport-for-development theories (64, 65). All these frameworks share the common feature of being contextually dependent, and having well-thought mechanisms leading to precise outcomes (55). In our case, our mechanism stand with emotions as ways to make the climate crisis visible to SDP participant, to then achieve the outcome of mobilizing climate activism and individual behaviour change.

**Figure 1:**
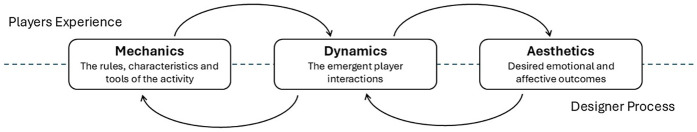
The Mechanics, Dynamics, and Aesthetics (MDA) framework.

In this section we introduce a framework that has gained popularity within the gaming industry and is concentrated into how games can be intentionally designed to achieve specific emotional outcomes. This framework is the mechanics-dynamics-aesthetics (MDA) framework (22, 23). The mechanics segment of MDA refers to the rules, components, systems, settings, and the overall resources that make up a given game. Mechanics refer to the “coded” parts of a game, which restrict, enable or guide a player within the game. Dynamics, resulting from a game's mechanics, encompass the player's emergent interactions and adopted strategies during gameplay. Aesthetics here refer to the desired emotional responses of a player from both the mechanics and dynamics. These can also be referred to as the player experience goals, that can range to a variety of different emotions, such as hope, awe, fellowship, and direction among others. Fundamentally, MDA is a powerful tool for designing experiences that intend to evoke specific feelings. These experiences are cultivated by carefully designing game mechanics to anticipate and guide player behaviour towards positive action (Figure 1).

More broadly, the use of MDA falls under the wider umbrella of using elements and theories of game design into non-game contexts, in a process that is popularly known as “gamification”. This process is often attached to education, and its ultimate objective is to make participants in each initiative feel like they are playing a game, while, in reality, they are fulfilling a higher purpose (66). This higher purpose is often one of education or behaviour change (67), just like for SDP initiatives. By immersing themselves into a game experience [often referred to as serious gaming (67);], players can learn or empathise with the key messages or teaching that the game is seeking to deliver.

For example, “Path Out” is a game created by a refugee and designed to empathise and engage people with the realities of the refugee journey, aiming to reduce prejudice and promote understanding (68). Games such as “Escape from Diab” and “Nanoswarm: Invasion from Inner Space” were purposefully designed to improve the diet of children, resulting in an increase of fruit and vegetables intake for their gamers. And, closely related to sustainability and ecological emotions, environmental videogames have also led to positive results (69). For example “Lumino City”, a sustainability themed puzzle adventure game, has shown to provide emotional engagement with the complex theme of resource management (70).

To specifically showcase the MDA framework at work, we can analyze UN's initiative using Minecraft for community participation (71). The UN-Habitat's initiative uses the videogame Minecraft as a participatory tool, enabling community members to collaboratively design and visualize improvements for their local public spaces. This approach aims to empower residents by giving them an accessible and engaging way to contribute to urban development.

The *mechanics* are here the foundational rules and tools: Minecraft itself, with its “digital Lego” building capabilities, creative mode, and multiplayer functionality, serves as the core engine. UN-Habitat augments this by providing a pre-built Minecraft model of the existing site, which acts as a tangible starting point, and a structured workshop process involving defined steps, expert facilitation, and dedicated time for design. These mechanics, including the inherent simplicity of block-based construction, are intentionally chosen to lower the barrier to entry for participants who may not be proficient with computers.

The *dynamics* stem from community members’ interactions with these mechanics, encompassing not just adherence to workshop instructions but also emergent strategies and run-time behaviours, such as collaborative problem-solving to translate abstract community needs into tangible block structures. They also negotiate design choices within their groups, leading to consensus or creative compromises. Finally, they also experiment with different spatial arrangements, iteratively refining their ideas based on the visual feedback from the Minecraft model and peer discussion. The dynamic of learning the controls of the game and then using that newfound skill to articulate complex urban design ideas is crucial. Furthermore, the act of presenting and justifying their models fosters dynamics of advocacy and persuasive communication. These dynamics, meaning the interplay of creation, negotiation, learning, and articulation within the game's and workshop's constraints, are what bridge the designed system to the intended experience.

Ultimately, the goal is to evoke specific *aesthetics*, or emotional responses. This use of Minecraft aims for fellowship, as participants work collaboratively, enhancing community cohesion. Discovery plays a role as participants explore new possibilities for their environment and learn from each other. The process also engenders a sense of challenge, in mastering the tool and working through design problems, leading to the feeling of empowerment when those challenges are overcome, and their ideas are visibly represented and considered. The playful, constructive nature of Minecraft can also tap into an aesthetic of positive fantasy (thus imagining a better future) and contribute to an overall feeling of hope and positive engagement in the community's development.

### Theorizing and exemplifying the MDA framework in SDP

Finally, we get to the application of the MDA framework in SDP, to design SDP interventions that can engage young people in climate action.

To purposefully address the emotional outcome of an SDP initiative, the MDA framework should be applied “backwards”, that is starting from the emotions that one is trying to elicit through the game design. Here we are trying to answer the question of how SDP can engage with the climate crisis and promote climate action (14, 49). Ultimately, the focus should be on using sport as a mean to create empathy for the environment, advancing feelings of hope and topophilia, which would eventually lead to further action. The dynamics, as a result, should focus on the engagement with play and fun, instead of competing. Prioritizing play over competition facilitates the emergence of solidarity (17, 19), a dynamic particularly crucial for fostering collective climate action (12). Furthermore, the way the activity is designed should indeed take into account both the interaction that players have with each other, but also the interactions that they have with the environment. Finally, in order to do this, we need to have mechanics, and therefore rules and resources, that involve the natural environment in a meaningful manner. Below, we offer an illustrative example of an SDP activity designed using the MDA framework to foster positive ecological emotions and encourage climate action. This conceptual example prioritises play, collaboration, and direct engagement with the local environment, moving away from purely competitive or overly structured traditional sport formats.

In this example, we could use an adaptation of sport, such as plogging, where participants must interact with the environment to complete the activity and simultaneously take action to clean their surrounding area (72). People should work in teams to clean an assigned zone, either at the same time or under a relay format. Focusing on the dynamics, each team does not compete with each other, but rather works towards achieving a collective win of a cleaner surrounding. The game can then end after a certain amount of litter is collected, and with a group discussion on the learnings and emotional outcomes of the activity.

**Figure 2:**
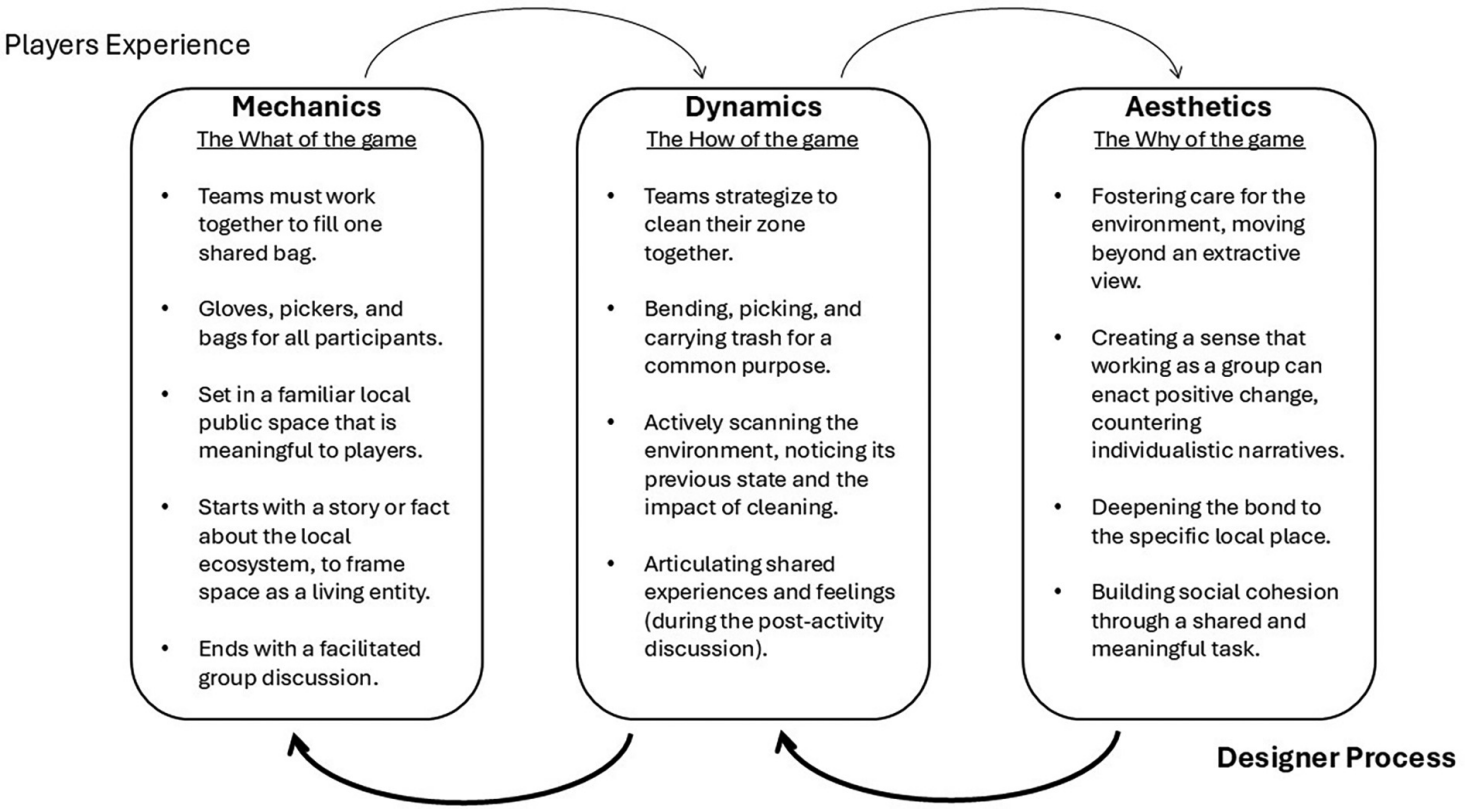
An application of the MDA framework to an SDP plogging activity.

From an MDA perspective, the aesthetic objective is that of achieving several emotional outcomes. First, fellowship and connection, as participants work collaboratively towards a common and tangible goal of cleaning. Second, empowerment and accomplishment, seeing the tangible impact of their action in a now cleaner environment. Third, topophilia and stewardship, by developing care and responsibility for a natural area through direct positive action. The dynamics to achieve such objectives could be multiple and varied. For example, teams could discuss how to best cover their zones, once again fostering collaboration. They will be paying attention to the natural environment, as there is a constant need to actively scan their surroundings to collect litter. They will also find further emotion in necessary embodied action, as the acts of bending, picking, carrying, and moving will connect them both to themselves and the environment. To do so, the mechanics will need to rely on the activity being set in an area that needs cleaning, as well as a safety briefing on how to handle litter and be aware of environmental hazards, further enhancing the learning experience of participants. Resources such as litter bags, gloves, and pickers, are also instruments that create further familiarity with taking care of the environment. A description of this example is further illustrated in Figure 2.

The example shows how the MDA framework of SDP works as an inverted pyramid, where we hold the objective of eliciting ecological emotions while using sport and physical activity as the main tool to do so. This application fulfils the need of think of SDP as an ecological endeavour (14), as all the components synergically work towards ecological outcomes while also greening the practices of the movement (16, 49). Furthermore, the focus on play over competition allows the fostering of emotions that are not only rooted in climate action, but also in solidarity and collectivism (19).

### Cautions and challenges

While presenting this conceptual exploration of game design within SDP programming is an important exercise to identify positive outcomes, it is critically important to reflect on potential and concrete challenges that exist in adopting such an approach.

First, designing to elicit an emotion from participants should come with ethical considerations. Indeed, the notion raises immediate ethical questions about potential manipulation vs. genuine facilitation. It is crucial that MDA is applied with a commitment to transparency and participant agency. This aspect is particularly important in relation to critiques advanced towards SDP on restraining freedoms (19). The aim should be to create conditions conducive to the emergence of certain emotions, rather than attempting to prescribe or coerce feelings. Facilitation should focus on opening spaces for reflection and authentic emotional expression, acknowledging that individual responses will vary. The emphasis, as argued in this paper, is largely on fostering positive, empowering emotions, but even then, ethical practice demands respect for individual affective experiences.

Second, there is a need to handle negative emotions constructively. While the intended focus is often on positive ecological emotions, experiences with environmental issues can also evoke challenging emotions like eco-anxiety, grief, or frustration. An MDA-designed programme must be prepared to acknowledge and constructively channel these emotions if they arise. Importantly, the distinction between positive and negative emotions needs to be contextually understood, and the contraddictions of this distinction acknowledged (8). For example, a dynamic of “shared concern” could be channelled into an aesthetic of “collective agency” if the activity provides an outlet for action. This requires skilled facilitation and a safe, supportive environment.

Third, the MDA framework cannot be thought of as a generalisable tool, and will need adaptation when engaging diverse groups (48). A decolonial approach to MDA means co-designing the Aesthetics *with* the community, rather than imposing them, ensuring the desired emotional outcomes are locally meaningful and challenge, rather than reinforce, dominant power structures. Initiatives should be designed to be accessible and culturally relevant to diverse participants, considering physical abilities, socio-economic backgrounds, gender identities, and cultural norms. For example, co-designing mechanics with community members, particularly those from marginalized groups, is a key decolonial practice that can ensure relevance and ownership (73). Similarly, it is vital to recognize that desired emotions might differ across cultures or groups, or be interpreted differently. For instance, what constitutes “empowerment” or “connection” can be highly contextual. A decolonial lens would question universalist assumptions about emotions and seek to understand and design for locally meaningful affective outcomes. MDA can be a tool for this if applied with cultural humility and a commitment to co-creation.

Fourth, there is a risk that a focus on game-like mechanics could lead to superficial engagement or greenwashing if the activities do not connect to genuine environmental issues or deeper learning. This relates to wider concerns of neoliberal logics within SDP, where nature and green messaging are once again used to put forward interests and agendas of powerful elites (62). The aesthetics aimed for must be substantive, fostering genuine shifts in understanding, care, or motivation, rather than just leading to amusement. The link between the activity and real-world ecological contexts and actions must be explicit and meaningful.

Finally, as noted by Giulianotti (16), a significant challenge is the environmental impact of sport and SDP itself. When designing SDP interventions for climate action using MDA, the “Mechanics” must inherently consider sustainability. This means prioritizing low-impact activities, using recycled or sustainable materials, choosing accessible local venues to minimize travel, and potentially incorporating elements that educate about, and reduce, the activity's own footprint. The plogging example that we have presented is quite strong in this regard, as the activity itself is environmentally positive.

## Conclusion

It is clear that to address the current climate crisis we need creative solutions that can engage (young) people into nurturing the environment. Inspired by recent calls to seek social alternatives over solutions to current problems, this paper has theorized how SDP can be designed to be an imaginative space of learning where positive emotions can be nurtured for environmental change. It has done so by borrowing theoretical ideas from the world of game design and applying them to sport. As a conceptual paper, this study comes with the limitation that these ideas are not empirically tested, yet comes with the ambition to continue a nascent discussion on how SDP can learn from engaging with multiple disciplines (24), and areas of study (46).

The implications for SDP organizations largely concern program design. Initiatives must consider emotional outcomes as powerful vehicles of social transformation, with sport being an exceptional emotional platform to mobilise and empower people (12). The MDA framework that we here propose can guide the design of such initiatives. Practitioners should use MDA “in reverse” (Aesthetics, Dynamics, Mechanics; see Figure 2) as a systematic process for designing or adapting sport-based activities. Starting with the desired ecological emotions (Aesthetics), they can then brainstorm the types of interactions and behaviours (Dynamics) that would evoke these feelings. Finally, they can devise the rules, resources, and environmental set-up (Mechanics) that would facilitate these dynamics. This structured approach can help move beyond vague goals like raising awareness (14), to more specific, measurable, and actionable affective objectives. Importantly, MDA provides a clear vocabulary, especially in relation to Aesthetics, for articulating programme goals related to emotion and climate. This can be invaluable for communication with participants, funders, and other stakeholders, making the intended affective impact explicit and central to the programme's theory of change, rather than an assumed by-product.

This paper proposes that “gaming” SDP can be a strategy to instigate behaviour change that positively affects the environment, and directs towards climate action. A significant challenge is that sport often treats nature as a mere backdrop or a resource to be consumed. A poorly designed activity, even one with good intentions, can reinforce this extractive view. The MDA framework offers a direct way to counter this. By prioritizing an aesthetic of stewardship and collectivism, over conquest or competition, it fundamentally alters the objectives of sport. The goal is no longer to use nature, but to interact with and care for it. Furthermore, SDP has been critiqued for its frequent focus on individual development, which can align with neoliberal agendas of self-improvement while ignoring systemic issues. This often fails to build the interactional empowerment needed for collective action. MDA provides a tool to design specifically for collective outcomes. By defining an aesthetic of solidarity, the practitioner is forced to devise mechanics that necessitate collaborative dynamics. Nonetheless, the application of MDA for design purposes should be done with caution. SDP activities should be designed with fundamental ethical principles, rooted in decolonial approaches and a commitment to achieving genuine and substantive change.

Similar implications apply to research. Most notably, future studies should assess and explore SDP interventions that aim to foster climate action, adopting a critical lens that evaluates the fostering of freedom and imagination within such initiatives, specifically in relation to the role of play (17, 18). Specifically, future studies should explore and validate the use of MDA as an SDP design tool. Furthermore, research should continue investigating cross-disciplinary approaches to SDP programming (24). In this study we have relied on game design, environmental physiology, and cultural sociology. This has allowed us to theorise new, creative, and impactful ways to engage communities, in tackling the climate crisis through the power of play and embodied experience. While empirically testing and evaluating such ideas is of significant importance, we also encourage researchers to continue working with theoretical imagination and to cross disciplinary boundaries to identify solutions and alternatives to rapidly-evolving social challenges.
